# Restraint stress increases hemichannel activity in hippocampal glial cells and neurons

**DOI:** 10.3389/fncel.2015.00102

**Published:** 2015-04-02

**Authors:** Juan A. Orellana, Rodrigo Moraga-Amaro, Raúl Díaz-Galarce, Sebastián Rojas, Carola J. Maturana, Jimmy Stehberg, Juan C. Sáez

**Affiliations:** ^1^Departamento de Neurología, Escuela de Medicina, Pontificia Universidad Católica de ChileSantiago, Chile; ^2^Laboratorio de Neurobiología, Centro de Investigaciones Biomédicas, Facultad de Ciencias Biológicas and Facultad de Medicina, Universidad Andres BelloSantiago, Chile; ^3^Departamento de Fisiología, Facultad de Ciencias Biológicas, Pontificia Universidad Católica de ChileSantiago, Chile; ^4^Instituto Milenio, Centro Interdisciplinario de Neurociencias de ValparaísoSantiago, Chile

**Keywords:** hemichannels, connexins, pannexins, stress, hippocampus, glia, neuron

## Abstract

Stress affects brain areas involved in learning and emotional responses, which may contribute in the development of cognitive deficits associated with major depression. These effects have been linked to glial cell activation, glutamate release and changes in neuronal plasticity and survival including atrophy of hippocampal apical dendrites, loss of synapses and neuronal death. Under neuro-inflammatory conditions, we recently unveiled a sequential activation of glial cells that release ATP and glutamate via hemichannels inducing neuronal death due to activation of neuronal NMDA/P2X_7_ receptors and pannexin1 hemichannels. In the present work, we studied if stress-induced glia activation is associated to changes in hemichannel activity. To this end, we compared hemichannel activity of brain cells after acute or chronic restraint stress in mice. Dye uptake experiments in hippocampal slices revealed that acute stress induces opening of both Cx43 and Panx1 hemichannels in astrocytes, which were further increased by chronic stress; whereas enhanced Panx1 hemichannel activity was detected in microglia and neurons after acute/chronic and chronic stress, respectively. Moreover, inhibition of NMDA/P2X_7_ receptors reduced the chronic stress-induced hemichannel opening, whereas blockade of Cx43 and Panx1 hemichannels fully reduced ATP and glutamate release in hippocampal slices from stressed mice. Thus, we propose that gliotransmitter release through hemichannels may participate in the pathogenesis of stress-associated psychiatric disorders and possibly depression.

## Introduction

Major depression disorder (MDD) is a disabling illness that adversely affects subject’s family, behavior, mood, activity and physical health. In developed countries, around 3% of MDD patients commit suicide, whereas several studies show that around 60% of all suicide victims had previously suffered from MDD (Arsenault-Lapierre et al., 2004). Interestingly, ample evidence indicates that stressful life events increase the risk for MDD, including acute and chronic stress (Kessler, 1997; Kendler, 1998; Hammen, 2005; Hammen et al., 2009). The term stress defines all physiological and/or psychological responses to events that require behavioral adjustment to overcome them (Sorrells et al., 2009; Popoli et al., 2011). Acute stress includes adaptive mechanisms necessary for survival, while chronic stress induces over-activation and dysfunction of stress-activated systems, resulting in further brain damage and depressive-like behavior (Sorrells et al., 2009; Popoli et al., 2011).

Restraint stress impairs both spatial hippocampal-dependent memory (Luine et al., 1994; Kleen et al., 2006) and hippocampal long-term potentiation (LTP; Pavlides et al., 2002; Alfarez et al., 2003). Such effects have been associated to retraction of apical dendrites as well as loss of synapses in the CA3 subregion of the hippocampus (Magariños and McEwen, 1995; Magariños et al., 1997). A proposed explanation is that these changes may be associated with dysregulated release of glutamate and NMDA receptor dysfunction (McEwen, 1999). Congruent with this idea, enhanced glutamate release in response to stress has been described (Gilad et al., 1990; Lowy et al., 1993), while NMDA but not AMPA receptors are reportedly involved in stress-related morphological changes in the hippocampus (Magariños and McEwen, 1995). Recently, we showed that amyloid-β peptide induces glutamate and ATP release via glial cell hemichannels, enhancing cell neuronal death by activation of NMDA/P2X_7_ receptors (Orellana et al., 2011a,b). In the central nervous system (CNS), gliotransmitter release is in part mediated by the opening of hemichannels formed by connexins or pannexins (Wang et al., 2013b). These unopposed membrane channels serve as aqueous pores permeable to ions and small molecules, providing a diffusional pathway of exchange between intra- and extracellular compartments. In glial cells, hemichannels allow the release of gliotransmitters that are necessary for different brain functions including glucosensing (Orellana et al., 2012), ischemic tolerance (Lin et al., 2008), fear memory consolidation (Stehberg et al., 2012), neuron-glia crosstalk (Torres et al., 2012) and chemoreception (Huckstepp et al., 2010). However, several independent studies have pointed out that onset and progression of homeostatic imbalances observed during neurodegeneration could be associated to enhanced hemichannel activity in the CNS (Takeuchi et al., 2006; Thompson et al., 2008; Karpuk et al., 2011; Orellana et al., 2011a,b; Gulbransen et al., 2012; Burkovetskaya et al., 2014).

Stress activates microglia (Tynan et al., 2010), which release glutamate and/or ATP via hemichannels (Shijie et al., 2009; Sáez et al., 2013), whereas proinflammatory cytokines released by activated microglia enhance hemichannel activity of astrocytes (Orellana et al., 2011b). Astroglial hemichannels in turn mediate the release of gliotransmitters (Orellana and Stehberg, 2014), which are critical for synaptic transmission and plasticity (Perea et al., 2009). Thus, stress may alter glial cell hemichannel activity, leading to important alterations in neuronal networking and possibly contributing to stress-induced functional and morphological changes in neurons. Therefore, we decided to investigate whether stress modulates the functional activity of hemichannels in glial cells and neurons in the hippocampus. Here, restraint stress is shown to increase differentially the opening of hemichannels in glial cells and neurons depending on the restraint protocol. Interestingly, these responses were associated with increased release of glutamate and ATP through these channels.

## Materials and Methods

### Reagents and Antibodies

Gap26, TAT-L2 and ^10^panx1 peptides were obtained from Genscript (New Jersey, USA). HEPES, DMEM, DNAse I, poly-L-lysine, CPP, A74003, MRS2179, brilliant blue G (BBG), oATP, ethidium (Etd) bromide, and probenecid (Prob) were purchased from Sigma-Aldrich (St. Louis, MO, USA). Fetal calf serum (FCS) was obtained from Hyclone (Logan, UT, USA). Penicillin and streptomycin were obtained from Invitrogen (Carlsbad, CA, USA). Normal goat serum (NGS) was purchased from Zymed (San Francisco, CA, USA). Cx43^E2^, a Cx43 hemichannel antibody to the second extracellular loop was kindly provided by Dr. Jean Jiang, Department of Biochemistry, University of San Antonio, USA (Siller-Jackson et al., 2008).

### Animals and Restraint Stress Protocols

All animal experimentation were conducted in accordance with the guidelines for care and use of experimental animals of the National Institute of Health (NIH) and local guidance documents generated by the *ad hoc* committee of the Chilean Research Organization (CONICYT). The studies were performed according to protocols approved by the bioethical committee of Universidad Andrés Bello, Chile.

Wild-type C57BL/6 or Panx1^−/−^ male mice weighting between 25 and 35 g were used. The generation of Panx1^−/−^ (KO) mice has been described previously (Anselmi et al., 2008). Mice were housed individually in plastic homecages in a temperature controlled room at 24°C, under a 12 h:12 h illumination cycle (lights on at 8:00 AM). All animals were kept in individual cages throughout the study and had ad libitum access to standard rodent food pellets and tap water. Animals were maintained under standard laboratory conditions for at least 2 weeks before starting the stress protocol. To stress animals, we used a modified version of the restraint protocol described by Mozhui et al. (Mozhui et al., 2010). Animals were segregated in three groups: acute stress, chronic stress and control. For acute stress, animals were placed in ventilated 50 ml Falcon tubes only once for 2 h, prior to behavioral tests. For chronic stress, each mouse was placed into a tube for 2 h per day (14:00 P.M. to 16:00 P.M.) for 10 consecutive days before behavioral evaluations. Non-restrained mice (control group) remained in the home cage until behavioral evaluations.

### Behavioral Evaluations

#### Open Field Test

Thigmotaxis was evaluated in the open field test, as reported previously (Takemoto et al., 2008; Ito and Ito, 2011). Animals were placed in the central zone of a plexiglas rectangular box (40 × 60 × 60 cm) and allowed to explore for 5 min, while being recorded digitally for subsequent off-line analysis. For analysis, the recorded trial was analyzed by a blinded investigator and the floor of the open field was virtually divided in the screen into 10 × 10 cm squares. Time spent in the periphery (thigmotaxis) and time spent in the center of the open field were measured.

#### Dark and Light Exploration Test

This test was performed as reported elsewhere (Crawley, 1981; Mathis et al., 1995). The dark and light box consisted of a plexiglas apparatus (50 × 30 × 20 cm) separated by two compartments: one dark (lacking illumination) with black walls (20 × 15 × 20 cm) and one lit compartment with transparent walls. Both compartments were connected by a small opening (6 × 6 cm) at the floor level. The lit compartment was brightly illuminated (~1000 Lux) by a lamp from above. Mice were placed on the lit compartment looking opposite to the dark compartment and allowed to freely explore the apparatus for 5 min. Difference between total time in the lit compartment and the latency to enter the dark compartment for the first time was measured and plotted as “time in the lit” compartment. All trials were recorded digitally for subsequent off-line analysis by a blinded investigator.

### Acute Hippocampal Slices

Mice were decapitated and brains were dissected and placed in ice-cold artificial cerebral spinal fluid (ACSF) containing (in mM): 125 NaCl, 2.5 KCl, 25 glucose, 25 NaHCO_3_, 1.25 NaH_2_PO_4_, 2 CaCl_2_, and 1 MgCl_2_, bubbled with 95% O_2_/5% CO_2_, pH 7.4. Hippocampal coronal slices (400 µm) were cut using a vibratome (Leica, VT 1000GS; Leica, Wetzlar, Germany) filled with ice-cold ACSF. The slices were transferred at room temperature (20–22°C) to a holding chamber and immersed in oxygenated ACSF, pH 7.4, for a stabilization period of 30 min before using them.

### Dye Uptake and Confocal Microscopy

For “snapshot” experiments, acute slices were incubated with 100 µM Etd for 15 min in a chamber oxygenated by bubbling gas mixture (95% O_2_ and 5% CO_2_) into ACSF, pH 7.4. Slices were then washed five times with ACSF, fixed at room temperature with 4% paraformaldehyde for 30 min, rinsed extensively in PBS and stored overnight at 4°C in a cryoprotectant solution (30% sucrose). Next day, slices were frozen and dissected in 12–16 µm-thick sections with a cryostat. The sections were then mounted in Fluoromount and incubated in 0.1% PBS-Triton X-100 containing 10% NGS for 30 min. Afterwards, sections were incubated overnight at 4°C with either anti-Iba-1 polyclonal antibody (1:300, Wako), anti-GFAP monoclonal antibody (1:300, Sigma) or anti-NeuN monoclonal antibody (1:400, Chemicon) to detect microglia, astrocytes or neurons, respectively. All antibodies were diluted in 0.1% PBS-Triton X-100 with 2% NGS. After five rinses in 0.1% PBS-Triton X-100, sections were then incubated for 1 h at room temperature with goat anti-rabbit Alexa Fluor 488 (1:1,500), goat anti-mouse Alexa Fluor 488 (1:1,500) or goat anti-mouse Alexa Fluor 647 (1:1,500) antibody. After several washes, slices were mounted in Fluoromount, coverslipped and examined in a confocal laser-scanning microscope (Olympus Fluoview FV1000, Tokio, Japan). Stacks of consecutive confocal images taken with a 63 X objective at 500 nm intervals were acquired sequentially with two lasers (argon 488 nm and helium/neon 543 nm), and Z projections were reconstructed using Fluoview software. Dye uptake ratio was calculated as the subtraction (F−F0) between the fluorescence (F) from respective cell and the background fluorescence (F0) measured where no labeled cells were detected. At least six cells per field were selected from at least three fields in each hippocampal slice.

### Measurement of Extracellular ATP and Glutamate Concentration

Acute hippocampal slices were immersed in oxygenated ACSF (as above), pH 7.4, at room temperature (20–22°C) for 30 min under control conditions or exposed to different agents. Then, extracellular ATP was measured using a luciferin/luciferase bioluminescence assay kit (Sigma-Aldrich), while extracellular levels of glutamate were determined using an enzyme-linked fluorimetric assay (Sigma-Aldrich). The amount of glutamate and ATP in each sample was inferred from standard curves as described previously (Orellana et al., 2011a,b). Briefly, after the experiments, the slices were washed twice with ACSF solution and sonicated in ice-cold PBS containing 5 µM EDTA, Halt (78440) and T-PER protein extraction cocktail (78510) according to manufacturer instructions (Pierce, Rockford, IL). Total proteins from tissue homogenates were measured using the Bio-Rad protein assay.

### Data Analysis and Statistics

For each data group, results were expressed as mean ± standard error (SEM); *n* refers to the number of independent experiments. For statistical analysis, each treatment was compared with its corresponding control, and significance was determined using a one-way ANOVA followed, in case of significance, by a Tukey *post hoc* test.

## Results

### Restraint Stress Enhances Cx43 and Panx1 Hemichannel Activity in Brain Cells

**Figure 1:**
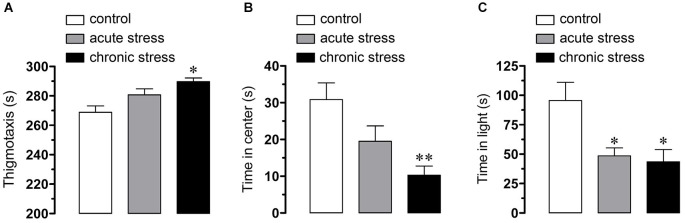
**Behavioral indicators of stress**. Averaged data of anxiety-like symptoms as assessed by thigmotaxis **(A)** and time in the center **(B)** of the open field as well as time in the lit compartment of the dark and light box **(C)** in mice under control conditions (white bars), after acute (gray bars) or chronic (black bars) restraint stress. *p < 0.05; **p < 0.01. Averaged data were obtained from seven animals for each group.

Restrain stress impairs hippocampus-dependent spatial memory and hippocampal synaptic plasticity, inducing LTP deficits (Luine et al., 1994; Pavlides et al., 2002; Alfarez et al., 2003; Kleen et al., 2006), whereas hemichannel opening has been linked to glial and neuronal dysfunction (Takeuchi et al., 2006; Orellana et al., 2011a,b, 2013; Shestopalov and Slepak, 2014). Therefore, we investigated whether restrain stress affects the functional activity of hemichannels in hippocampal microglia, astrocytes and neurons. Anxiety-like symptoms increased as result of restraint stress using different models. A non-significant tendency for increased thigmotaxis was found in animals that underwent acute restraint stress, increment that became significant in mice subjected to chronic restraint stress, when compared to control mice (from 268.3 ± 4.3 s to 280.6 ± 2.7 s and 289.7 ± 2.7 s, respectively, *n* = 7, *p* < 0.05) (Figure 1A). Moreover, in the open field test, the time spent in the center was significantly reduced in mice subjected to acute restraint stress compared to control mice, a decrease that was even larger after chronic restraint stress (from 30.8 ± 4.6 s to 19.5 ± 3.8 s and 10.3 ± 2.7 s, respectively, *n* = 7, *p* < 0.05) (Figure 1B). In addition, in the dark and light exploration test, both acute and chronic restraint stress induced a significant reduction in the time spent in the lit compartment compared to control mice (from 95.5 ± 15.5 s to 48.5 ± 7 s and 43.4 ± 11.2 s, respectively, *n* = 7, *p* < 0.05) (Figure 1C). These results are indicative of anxiety-like symptoms in mice subjected to acute and chronic restrain confirming that they were stressed and suggesting that chronic stress induces more anxiety-like symptoms than acute stress.

**Figure 2:**
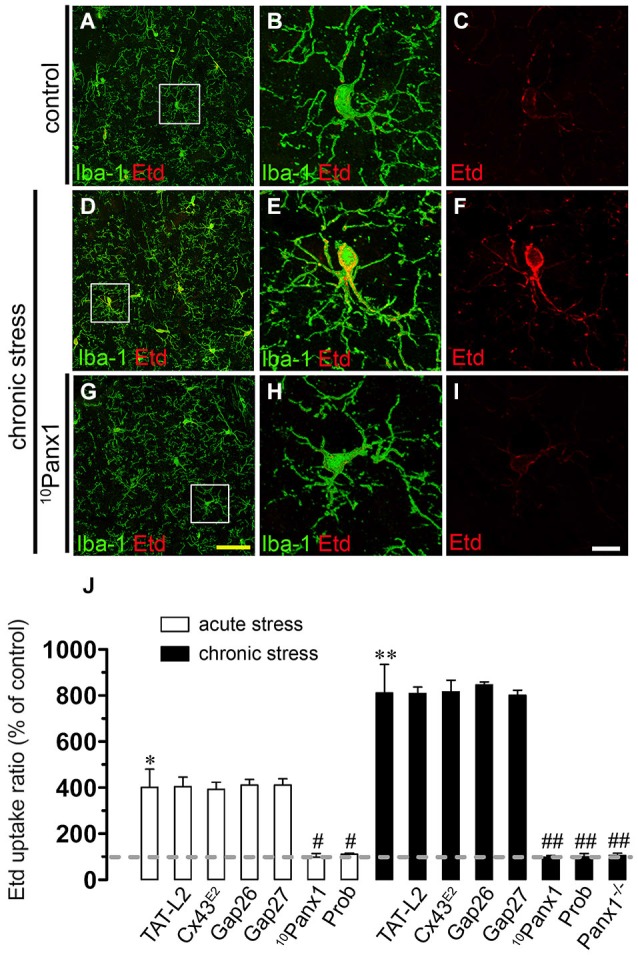
**Restraint stress increases Panx1 hemichannel activity in microglia**. Representative images showing Iba-1 (green) and Etd (red) uptake of acute hippocampal brain slices from control mice **(A–C)** and mice subjected to chronic restraint stress **(D–F)**. In addition, the effect of ^10^panx1 on Etd uptake is shown in mice subjected to chronic restraint stress **(G–I)**. Images of hippocampal microglia were taken from the area depicted within the white square in panels **(A,D)** and **(G)**. **(J)** Averaged data of Etd uptake ratio normalized to control conditions (dashed line) of microglia from mice subjected to acute (white bars) and chronic (black bars) restraint stress. Also shown are the effects of the following blockers applied during the Etd uptake essay: TAT-L2 (100 µM), Cx43^E2^ (1:500), Gap26 (100 µM), Gap27 (100 µM), ^10^panx1 (100 µM) or probenecid (Prob, 500 µM). Also it is shown data of microglia of hippocampal slices from Panx1^−/−^ mice. *p < 0.05, **p < 0.01, for the effect of restraint stress compared to control conditions; #*p* < 0.05, ##*p* < 0.01, effect of each blocker compared to the respective effect induced by restraint stress. Averaged data was obtained from three independent experiments. Calibration bars: yellow bar = 70 µm; white bar: 10 µm.

**Figure 3:**
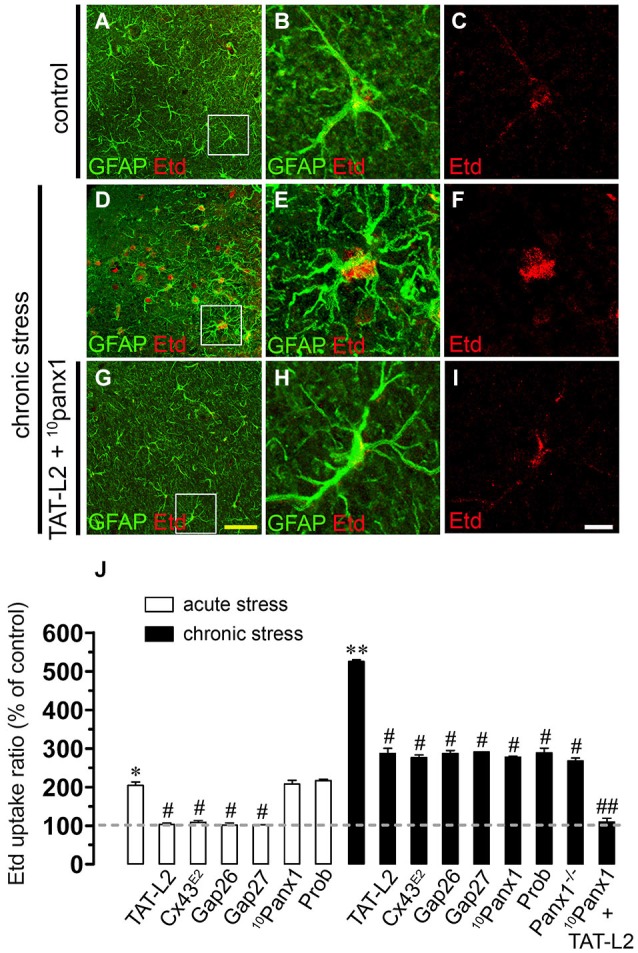
**Restraint stress increases Cx43 and Panx1 hemichannel activity in astrocytes**. Representative images showing GFAP (green) and Etd (red) uptake of acute hippocampal slices made from control mice **(A–C)** and mice subjected to chronic restraint stress **(D–F)**. In addition, the effects of TAT-L2 and ^10^panx1 on Etd uptake was tested in mice subjected to chronic restraint stress **(G–I)**. Images of hippocampal astrocytes were taken from the area depicted within the white square in panels **(A,D)** and **(G)**. **(J)** Averaged data for Etd uptake ratio normalized to control conditions (dashed line) of astrocytes from mice subjected to acute (white bars) and chronic (black bars) restraint stress. Also shown are the effects of the following blockers applied during the Etd uptake essay: TAT-L2 (100 µM), Cx43^E2^ (1:500), Gap26 (100 µM), Gap27 (100 µM), ^10^panx1 (100 µM) or probenecid (Prob, 500 µM). Also it is shown data of astrocytes of hippocampal slices from Panx1^−/−^ mice. *p < 0.05, **p < 0.01 for the effect of restraint stress compared to control conditions; #*p* < 0.05, ##*p* < 0.01, effect of each blocker compared to the respective effect induced by restraint stress. Averaged data are obtained from three independent experiments. Calibration bars: yellow bar = 150 µm; white bar: 20 µm.

**Figure 4:**
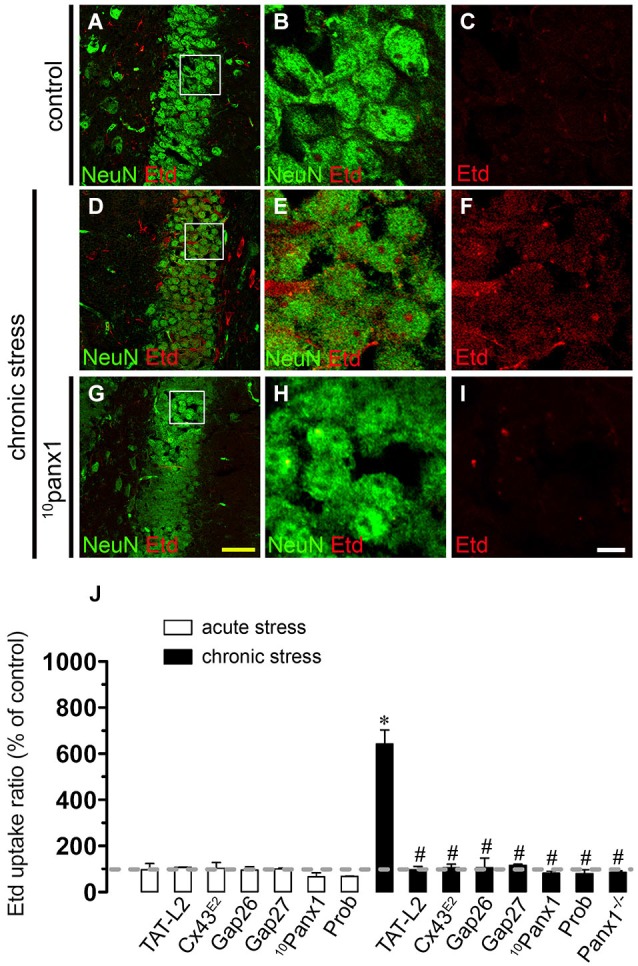
**Restraint stress increases Panx1 hemichannel activity in pyramidal neurons**. Representative images showing NeuN (green) and Etd (red) uptake of acute hippocampal brain slices from control mice **(A–C)** and mice subjected to chronic restraint stress **(D–F)**. In addition, the effect of ^10^panx1 on Etd uptake was tested in mice subjected to chronic restraint stress **(G–I)**. Images of hippocampal neurons were taken from the area shown within the white square in panels **(A,D)** and **(G)**. **(J)** Averaged data of Etd uptake ratio normalized to control conditions (dashed line) of neurons from mice subjected to acute (white bars) and chronic (black bars) restraint stress. Also shown are the effects of the following blockers applied during the Etd uptake essay: TAT-L2 (100 µM), Cx43^E2^ (1:500), Gap26 (100 µM), Gap27 (100 µM), ^10^panx1 (100 µM) or probenecid (Prob, 500 µM). Also it is shown data of neurons of hippocampal slices from Panx1^−/−^ mice. *p < 0.05; effects of restraint stress compared to control conditions; #*p* < 0.05, effects of each blocker compared to the respective effect induced by restraint stress. Averaged data are obtained at from three independent experiments. Calibration bars: yellow bar = 80 µm; white bar: 10 µm.

To address whether restrain stress affects hemichannel activity of brain cells, Etd uptake was measured in hippocampal slices of mice that underwent each experimental condition. Etd crosses the plasma membrane of healthy cells passing through poorly selective channels, including connexin and pannexin hemichannels (Schalper et al., 2008). Upon binding to intracellular nucleic acids, Etd becomes fluorescent, and inhibition of this signal with specific blockers is indicative of dye uptake through hemichannels (Schalper et al., 2008; Sáez and Leybaert, 2014). Etd uptake was evaluated in “snapshot” experiments in Iba-1-positive microglia, GFAP-positive astrocytes and NeuN-positive neurons in hippocampal slices. All three cell types from control mice showed a low Etd uptake ratio (Figures 2A–C, 3A–C, 4A–C) as demonstrated previously (Karpuk et al., 2011; Orellana et al., 2011b). However, acute restraint stress increased drastically the amount of Etd uptake in microglia and astrocytes (401 ± 78.6% and 204.4 ± 8.9%; respectively, compared to control, *n* = 3, *p* < 0.05) (Figures 2J, 3J), but not in pyramidal neurons (Figure 4J). Microglia have been shown to express functional unopposed pannexin1 (Panx1) and connexin43 (Cx43) hemichannels (Orellana et al., 2011b, 2013; Sáez et al., 2013). The possible role of Panx1 hemichannels in acute stress-evoked Etd uptake was studied using probenecid and the mimetic peptide ^10^panx1 with an amino acid sequence homologous to the second loop of Panx1 (Pelegrin and Surprenant, 2006; Silverman et al., 2008). Probenecid (500 µM) and ^10^panx1 (200 µM) nearly abolished the increase in microglial Etd uptake triggered by acute restraint stress (from 401 ± 78.6% to 110.3 ± 5.9% and 98.3 ± 15.9%, respectively, *n* = 3, *p* < 0.005) (Figures 2G–J). In contrast, mimetic peptides homologous to the cytoplasmic (TAT-L2), first (Gap26) or second (Gap27) extracellular loop of Cx43 (Wang et al., 2013a) and a Cx43 hemichannel antibody (Cx43^E2^) (Siller-Jackson et al., 2008), did not affect acute stress-induced Etd uptake by microglia (Figure 2J). Astrocytes express functional unopposed hemichannels formed by Cx43 (Contreras et al., 2002) and Panx1 (Iglesias et al., 2009), thereby we used TAT-L2, Cx43^E2^, Gap26, probenecid and ^10^panx1 to determine the contribution of each channel type in acute stress-induced Etd uptake in astrocytes. TAT-L2, Cx43^E2^, Gap26 and Gap27 fully reduced astroglial cell Etd uptake evoked by acute restraint stress (from 204.4 ± 8.9% to 103 ± 3.8%; 108.4 ± 4.5%, 101.4 ± 5.9% and 101.8 ± 0.3%, respectively, *n* = 3, *p* < 0.005) (Figures 3G–J). In contrast, ^10^panx1 and probenecid did not affect the stress-induced Etd uptake (Figure 3J).

Responses to acute stress are generally adaptive, but long lasting stress can cause persistent changes and even irreversible damage (Millán et al., 1996; Dhabhar and McEwen, 1997). In agreement with this notion, we found that Etd uptake (% compared to control conditions) induced by chronic stress in microglia and astrocytes was stronger than that found after acute stress (401 ± 78.6% vs. 811.1 ± 124.1%; respectively; and 204.4 ± 8.7% vs. 525.3 ± 4.5%; respectively; *n* = 3, *p* < 0.05) (Figures 2D–F,J, 3D–F,J). Probenecid and ^10^panx1 nearly abolished the increase in microglial cell Etd uptake triggered by chronic restraint stress (from 811.1 ± 124.1% to 100.9 ± 13.9% and 97.5 ± 7.7%, respectively, *n* = 3, *p* < 0.005) (Figure 2J), whereas TAT-L2, Cx43^E2^, Gap26 and Gap27 failed to affect this response (Figure 2J). The above findings suggest that in microglia, Panx1 but not Cx43 hemichannels, mediate the restraint stress-induced Etd uptake. This interpretation was supported by the absence of chronic stress-induced microglia hemichannel activation in hippocampal slices from Panx1^−/−^ mice (Figure 2J). On the other hand, TAT-L2, Cx43^E2^, Gap26 and Gap27 partially reduced astroglial Etd uptake evoked by chronic restraint stress (from 525.3 ± 4.6% to 287 ± 13.1%; 276.2 ± 13.1%, 286 ± 7.7% and 290.8 ± 0.6%, respectively, *n* = 3, *p* < 0.05) (Figure 3J). Moreover, contrary to the results observed in astrocytes from acute stress mice, ^10^panx1 and probenecid inhibited prominently the chronic stress-induced Etd uptake (from 525.3 ± 4.6% to 277.3 ± 2.5% and 288.6 ± 12.1% respectively, *n* = 3, *p* < 0.005) (Figure 3J). These data were in agreement with the fact that chronic stress triggered a partial increase of astroglial hemichannel activity in hippocampal slices from Panx1^−/−^ mice (Figure 3J). Moreover, consistent with the idea that both Cx43 and Panx1 hemichannels were the main contributors to chronic stress-induced Etd uptake in astrocytes, simultaneous blockade of these channels with TAT-L2 and ^10^panx1 fully reduced the response (from 525.3 ± 4.6% to 109.2 ± 9.8%, respectively, *n* = 3, *p* < 0.005) (Figure 3J). In contrast to the lack of effect of acute stress on neuronal hemichannel activity, chronic stress evoked a prominent increase on Etd uptake in pyramidal neurons (641.9 ± 61.7%, *n* = 4) (Figures 4A–F). Since most available evidence support the notion that neurons express hemichannels formed by Panx1 (Thompson et al., 2006), we used ^10^panx1 and probenecid to determine the contribution of these channels on chronic restraint stress-induced neuronal Etd uptake. ^10^panx1 and probenecid strongly reduced the stress-induced Etd uptake observed in pyramidal neurons (from 641.9 ± 61.7% to 81.8 ± 11% and 80.1 ± 17%, respectively, *n* = 3, *p* < 0.005) (Figures 4G–J), whereas TAT-L2, Gap19 and Gap26 caused a similar inhibition (from 641.9 ± 61.7% to 96.5 ± 14.9%; 105.1 ± 15.4% and 106 ± 41.5%, respectively, *n* = 3, *p* < 0.005) (Figure 4J). Accordingly, chronic restraint stress failed on evoke Etd uptake in hippocampal neurons from Panx1^−/−^ mice (Figure 4J). Moreover, basal levels of Etd uptake in microglia, astrocytes or neurons from Panx1^−/−^ mice were similar to that observed in wild type mice (data not shown). Overall, these data indicate that both acute and chronic restraint stress increase hemichannel opening of glial cells and neurons, being chronic restraint much more powerful than acute restraint stress in evoking this response.

### Chronic Restraint Stress Increase Panx1 Levels in Astrocytes and Neurons

**Figure 5:**
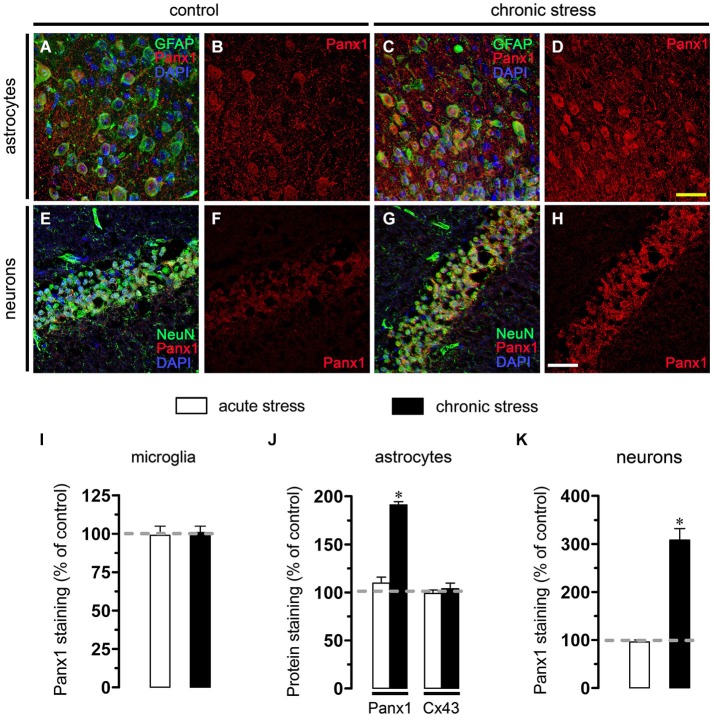
**Chronic restraint stress enhance Panx1 levels in astrocytes and neurons. (A–H)** Representative images showing Panx1 (red) and DAPI (blue) staining in GFAP positive astrocytes (green, **A–D**) and NeuN positive neurons NeuN (green, **E–H**) of acute hippocampal slices made from control mice (left panels) or subjected to chronic restraint stress (right panels). Calibration bars: yellow = 70 µm and white = 110 µm. **(I–K)** Averaged data of Panx1 and Cx43 staining normalized to control conditions (dashed line) in microglia **(I)**, astrocytes **(J)** and neurons **(K)** of hippocampal slices from mice subjected to acute (white bars) or chronic restraint stress (black bars). *p < 0.05, effect of chronic restraint stress compared with control conditions. Averaged data were obtained from three independent experiments.

Given that pathological conditions affect the expression of connexins and pannexins in the CNS (Rouach et al., 2002; Orellana et al., 2009), we examined whether chronic or acute restraint stress could modulate Cx43 and Panx1 levels in brain cells by confocal analysis. Interestingly, chronic but not acute restraint stress evoked a significant increase on Panx1 levels in astrocytes and neurons when compared to control conditions (Figures 5A–H,J,K). However, neither Cx43 nor Panx1 levels were affected in microglia in mice subjected to chronic restraint stress (Figure 5I). Similarly, for all tested conditions, Cx43 remained unchanged in astrocytes (Figure 5J).

### Hemichannel Opening Evoked by Chronic Restraint Stress Depends on Glutamatergic/Purinergic Signaling

**Figure 6:**
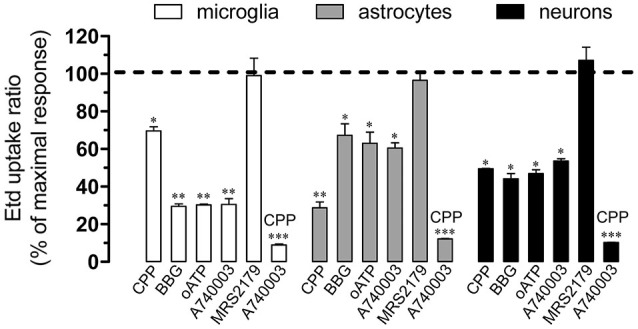
**Cx43 and Panx1 hemichannel activity evoked by chronic stress on NMDA/P2X_7_ receptor signaling**. Averaged data normalized to maximal Etd uptake ratio (dashed line) obtained after chronic restraint stress in microglia (white bars), astrocytes (gray bars) and neurons (black bars) treated with the following blockers during the Etd uptake essay: CPP (20 µM), BBG (10 µM), oATP (200 µM), A740003 (10 µM), MRS2179 (10 µM) or CPP (20 µM) plus MRS2179 (10 µM). *p < 0.05, **p < 0.01; ***p < 0.001, effect of each blocker compared to the respective effect induced by chronic restraint stress. Averaged data were obtained from four independent experiments.

Under activated state, glial cells release relevant amounts of gliotransmitters including glutamate and ATP, which underlie glia-to-glia and glia-to-neuron communication via glutamatergic and purinergic receptors, respectively (Perea et al., 2009; Perea and Araque, 2010). Because opening of hemichannels has been asociated with purinergic and glutamatergic signaling (Locovei et al., 2006; Thompson et al., 2008; Orellana et al., 2011a,b), we examined if NMDA and P2X_7_ receptors are involved in chronic restraint stress-induced Etd uptake. We found that CPP, a NMDA receptor blocker, strongly abolished the Etd uptake evoked by chronic restraint stress in astrocytes (from 100% of stress-induced effect to 28.7 ± 5.4%, *n* = 3, *p* < 0.05) (Figure 6), whereas in microglia and pyramidal neurons caused a small inhibition (from 100% of stress-induced effect to 69.7 ± 3.8% and 49.5 ± 0.4%, respectively, *n* = 3) (Figure 6). Moreover, blockade of P2X_7_ receptors with BBG, oATP and A740003 induced a prominent reduction on chronic stress-induced Etd uptake in microglia (from 100% of stress-induced effect to 29.6 ± 2.2%, 30.3 ± 0.7% and 30.5 ± 5.6%, respectively, *n* = 3, *p* < 0.05) and in neurons (from 100% of stress-induced effect to 44.2 ± 4.7%, 47 ± 3.4% and 53.6 ± 2.2%, respectively, *n* = 3, *p* < 0.05), with a lesser but significant decrease in astrocytes (from 100% of stress-induced effect to 67.3 ± 10.5%, 63 ± 10.2% and 60.5 ± 4.9%, respectively, *n* = 3) (Figure 6). To elucidate if in addition to P2X_7_ receptors, metabotropic purinergic receptors might also be involved in chronic restraint stress-induced hemichannel opening, we used MRS2179, a blocker of P2Y_1_ receptors, which has been previously linked to hemichannel opening in the CNS (Orellana et al., 2012; Sáez et al., 2013). MRS2179 did not affect the Etd uptake induced by chronic restraint stress in all brain cells studied (Figure 6). In agreement with the idea that both NMDA and P2X_7_ receptors are involved in hemichannel opening induced by chronic restraint stress, blockade of both receptors with CPP and A740003, respectively, fully reduced this response in microglia, astrocytes and neurons (from 100% of stress-induced response to 8.0 ± 1.0%, 12.1 ± 0.4% and 10.3 ± 0.1%, respectively, *n* = 3, *p* < 0.05) (Figure 6). Taken together these data indicate that Etd uptake induced by chronic restraint stress depends on NMDA/P2X_7_ receptor signaling.

### Chronic Restraint Stress Induces Cx43 and Panx1 Hemichannel-Dependent Release of Glutamate and ATP in Brain Cells

**Figure 7:**
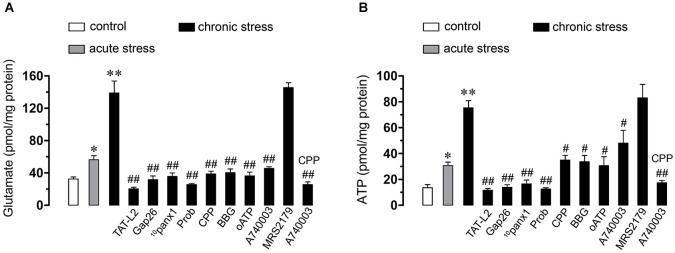
**Opening of Cx43 and Panx1 hemichannels allow the release of glutamate and ATP via NMDA/P2X_7_ receptor signaling**. Averaged data of glutamate **(A)** or ATP **(B)** release by hippocampal slices from mice under control conditions (white bars), subjected to acute (gray bars) or chronic (black bars) restraint stress. Also shown are the effects of the following blockers: TAT-L2 (100 µM), Gap26 (100 µM), ^10^panx1 (100 µM), probenecid (Prob, 500 µM), CPP (20 µM), BBG (10 µM), oATP (200 µM), A740003 (10 µM), MRS2179 (10 µM) or CPP (20 µM) plus MRS2179 (10 µM). *p < 0.05, **p < 0.01; effect of restraint stress compared to control conditions; #*p* < 0.05, ##*p* < 0.01, effect of each blocker compared to chronic restraint stress. Averaged data are obtained from four independent experiments.

Recently, gliotransmitters were shown to elicit their own release in an autocrine manner, via Cx43 and Panx1 hemichannels (Orellana et al., 2012, 2013). Given that NMDA/P2X_7_ receptors are involved in the Etd uptake observed in hippocampal cells of mice subjected to restraint stress, we evaluated whether this condition affects the glutamate and ATP release from hippocampal slices via Cx43 and/or Panx1 hemichannels. Acute and chronic stress strongly increased the release of glutamate and ATP (from 32.5 ± 4.4 pmol/mg to 56.4 ± 8.5 pmol/mg and 138.8 ± 25.9 pmol/mg, respectively and from 13.5 ± 4.2 pmol/mg to 30.7 ± 4.6 pmol/mg and 75.3 ± 9.6 pmol/mg, respectively, *n* = 3, *p* < 0.05) (Figure 7). Interestingly, TAT-L2, Gap26, ^10^panx1 and probenecid prominently reduced the release of glutamate (from 138.8 ± 25.9 pmol/mg to 20.4 ± 3.3 pmol/mg, 31.7 ± 7.8 pmol/mg, 35.7 ± 7.5 pmol/mg and 25.5 ± 2.3 pmol/mg, respectively, *n* = 3) and ATP (from 75.3 ± 9.6 pmol/mg to 11.6 ± 2.3 pmol/mg, 13.8 ± 3.6 pmol/mg, 16.5 ± 5.2 pmol/mg and 12.5 ± 1.8 pmol/mg, respectively, *n* = 3, *p* < 0.05) induced by chronic restraint stress (Figure 7). These findings indicate that chronic stress increases the release of glutamate and ATP via opening of Cx43 and Panx1 hemichannels.

In support for the notion that gliotransmitters can elicit their own release, we found that CPP, BBG, oATP, A740003, CPP plus A74003, but not MRS2179 abolished almost completely the release of glutamate (from 138.8 ± 25.9 to 38.7 ± 5.9 pmol/mg, 40.4 ± 7.9 pmol/mg, 36.3 ± 7.9 pmol/mg, 45.6 ± 3.5 pmol/mg, 25.6 ± 5.6 pmol/mg and 145.6 ± 10.5 pmol/mg, respectively, *n* = 3, *p* < 0.05) and ATP (from 75.3 ± 9.6 pmol/mg to 34.9 ± 6.5 pmol/mg, 33.7 ± 8.5 pmol/mg, 30.7 ± 11.9 pmol/mg, 48 ± 17.1 pmol/mg, 17.4 ± 3.2 pmol/mg and 83.1 ± 17.9 pmol/mg, respectively, *n* = 3, *p* < 0.005) induced by chronic restraint stress (Figure 7). This evidence suggests that both glutamate and ATP evoke their own release by an autocrine pathway possibly mediated by unopposed Cx43 and Panx1 hemichannels.

## Discussion

In this study, we showed that restraint stress increases the opening of Cx43 and Panx1 hemichannels in astrocytes; whereas Panx1 hemichannels are primarily activated in microglia and neurons. Moreover, the intensity of these responses depended on the duration of the restraint strees protocol and occurred by a mechanism linked to signaling via NMDA/P2X_7_ receptors. Furthermore, hemichannel opening induced by restraint stress troggered the release of both glutamate and ATP, two major gliotransmitters in the CNS.

Previous studies have demonstrated that restraint stress impairs spatial hippocampus-dependent cognitive performance (Luine et al., 1994; Kleen et al., 2006) and LTP (Pavlides et al., 2002; Alfarez et al., 2003) and induces glial cell activation (Nair and Bonneau, 2006; Sugama et al., 2007; Kwon et al., 2008). The present results suggest that at least part of the above mentioned changes induced by restraint stress could be explained by enhanced gliotransmitter release and further increase in extracellular gliotransmitter concentration within the CNS. It has been shown that gliotransmitter release through hemichannels underlies crucial functions of brain physiology (Lin et al., 2008; Huckstepp et al., 2010; Orellana et al., 2012; Stehberg et al., 2012; Torres et al., 2012). Nevertheless, several studies indicate that uncontrolled opening of these channels results in exacerbated release of gliotransmitters, which in high concentrations can be toxic to neighboring cells (Takeuchi et al., 2006; Orellana et al., 2011a,b). Now, we found that 2 h of restraint stress is sufficient to enhance opening of hemichannels in glial cells, whereas an enhanced response in neurons was achieved with a more prolonged restraint stress protocol (2 h for over 10 days). These results are in agreement with the fact that the consequences of physiological stress are usually adaptive in short term, but can be damaging when stress is chronic and long lasting (Millán et al., 1996; Dhabhar and McEwen, 1997).

In agreement with their surveillance role in the CNS (Block et al., 2007), microglia showed the highest changes on Etd uptake evoked by chronic restraint stress compared to astrocytes and neurons. Since this response was absent in hippocampal slices from Panx1^−/−^ mice and fully reduced by Panx1 blockers, hemichannels composed by the latter protein were mainly responsible of this phenomenon. In accordance with our results, recent studies have shown that pro-inflammatory conditions increase the opening of Panx1 channels in microglia (Orellana et al., 2013; Sáez et al., 2013). In our study, both astrocytes and neurons exhibited an evident increase in Etd uptake in mice subjected to chronic restraint stress as compared to control conditions. This response in astrocytes might be due to Cx43 and Panx1 hemichannels as mimetic peptides and blockers known to inhibit these channels (Pelegrin and Surprenant, 2006; Silverman et al., 2008; Wang et al., 2013a), completely inhibited the stress-induced Etd uptake. Another interpretation is that Panx1 hemichannels expressed by microglia or neurons could affect the opening of astroglial hemichannels by allowing the release of molecules that enhance their activity after restraint stress.

Neuronal Etd uptake induced by chronic restraint stress was strongly blocked by TAT-L2, Cx43^E2^, Gap26 or Gap27, ^10^panx1 and probenecid and absent in hippocampal slices from Panx1^−/−^ mice, indicating the involvement of Panx1 and Cx43 hemichannels. Neurons have been reported to express hemichannels formed by Panx1 and Cx36, but not Cx43 (Thompson et al., 2006; Schock et al., 2008; Orellana et al., 2011a). The fact that Cx43 hemichannel blockade reduced neuronal Etd uptake, suggests that astroglial Cx43 hemichannel activity constitutes a pre-requisite condition for the effects of chronic stress on neuronal hemichannels. Consistent with this, a recent study showed that gliatransmitter release via astroglial Cx43 hemichannels is required to trigger the amyloid-β peptide-dependent activation of Panx1 hemichannels in hippocampal neurons (Orellana et al., 2011b).

Glutamate and ATP are considered crucial transmitters on neuron-glia crosstalk and thereby their release through membrane proteins and vesicles is tightly regulated (Fields and Burnstock, 2006; Perea and Araque, 2010). In fact, high concentrations of glutamate and ATP at the synaptic cleft could be neurotoxic under pathological conditions (Lau and Tymianski, 2010; Arbeloa et al., 2012; Ashpole et al., 2013). As mentioned before, part of this neuronal damage could be the consequence of glutamate and ATP release via hemichannels (Takeuchi et al., 2006; Garré et al., 2010; Orellana et al., 2011a,b). Our findings indicate that chronic stress induced the release of hippocampal glutamate and ATP via Cx43 and Panx1 hemichannels, as their extracellular levels were reduced by TAT-L2, Cx43^E2^, Gap26 or Gap27, ^10^panx1 or probenecid.

What is the mechanism that underlies chronic stress-induced opening of Cx43 and Panx1 hemichannels? Previous studies have demonstrated that opening of these channels in glial cells relies on the rise of [Ca^2+^]_i_ linked to activation of NMDA, P2X_7_ or P2Y_1_ receptors (Orellana et al., 2011a,b, 2012; Sáez et al., 2013). Accordingly, in the present study Etd uptake and gliotransmitter release were both fully reduced by NMDA and P2X_7_ but not P2Y_1_ receptor blockers, suggesting that activation of Cx43 and Panx1 hemichannels likely occurs downstream in the NMDA/P2X_7_ pathway. Since activation of NMDA/P2X_7_ receptors raises [Ca^2+^]_i_ (Fields and Burnstock, 2006; Perea and Araque, 2010) and increased levels of [Ca^2+^]_i_ trigger gliotransmitter release via hemichannels (Locovei et al., 2006; Torres et al., 2012), it is plausible to suggest that stress induces NMDA/P2X_7_ receptor activation and further glutamate and ATP release via hemichannels. The latter subsequently evokes re-activation of those receptors to promote hemichannel-dependent release of these gliotransmitters.

Here, we observed that chronic but not acute restraint stress increases Panx1 levels in astrocytes and neurons, whereas the amount of Cx43 protein remained unchanged in all conditions and brain cells studied. Surface hemichannels account for ~11% of total Cx43 under resting conditions (Schalper et al., 2008), making them poorly detectable by immunofluorescence. Therefore, changes in Cx43 protein levels by immunodetection do not necessarily implicate change in surface hemichannels or in their activity, masked by a large amount of Cx43 forming gap junctions. Although it is still debated whether Panx1 hemichannels dock to form gap junctions (Sosinsky et al., 2011; Sahu et al., 2014), changes in Panx1 protein levels may reflect more surface hemichannels than in the case of Cx43. Thereof, it is possible that part of Etd uptake observed in astrocytes and pyramidal neurons could rely on the increase on surface levels of Panx1, whereas Cx43-dependent Etd uptake likely occurs via posttranslational modifications or changes in gating and sorting of Cx43 hemichannels (see previous paragraph). Further studies are required to elucidate whether changes in protein expression or degradation and sorting could contribute to the Cx43 and Panx1 hemichannel activity triggered by restraint stress.

Given the high expression of glucocorticoid (GC) receptors in the hippocampus, it may be one of the main target areas of GCs in the CNS (Popoli et al., 2011). During chronic restraint stress, blood and brain levels of GCs are persistently elevated, resulting in LTP and cognitive impairment and eventually promoting neuronal loss as well (Popoli et al., 2011). Moreover, both chronic stress and GCs increase glutamate levels (Moghaddam, 1993; Moghaddam et al., 1994) and [Ca^2+^]_i_ at hippocampal synapses (Elliott and Sapolsky, 1992, 1993). Taken altogether, we speculate that the chronic restraint stress protocol used in the present work increases GC brain levels, resulting in further activation of NMDA/P2X_7_ receptors in microglia and astrocytes. In agreement with this interpretation, chronic stress evokes NMDA receptor-dependent proliferation of microglia associated to GC receptor activation (Nair and Bonneau, 2006), whereas GC exposure primes cytokine release from microglia *ex vivo* (Frank et al., 2007). Furthermore, stress also activates astroglial cells (Kwon et al., 2008), while GCs enhances astrocytic [Ca^2+^]_i_ and ATP release (Simard et al., 1999). Further research is needed to unveil the exact mechanisms by which chronic stress affects hemichannels in glia and neurons and what the contribution of GCs on this process really is.

Although our working model does not recapitulate the mechanisms underlying the brain abnormalities induced by major depression and stress-associated psychiatric disorders, it allows us to dissect the specific contribution of hemichannels expressed by individual brain cell types. It must be noted that both chronic restraint stress and chronic GC administration are effective models for obtaining depressive-like symptoms in rodents (Levinstein and Samuels, 2014). In consequence, it is possible that hemichannel activation induced by chronic restraint stress may also contribute to the pathogenesis of depressive-like symptoms. Therefore, these findings may shed light into the early phases of neuronal dysfunction associated to stress, which may lead to major depression, post-traumatic stress disorder and other anxiety disorders. Our findings brings new vistas on the role of gliotransmitters on chronic stress and how hemichannels could arise as possible targets for developing novel pharmacological strategies to ameliorate different mental disorders associated to stress, anxiety and depression.

## Conflict of Interest Statement

The authors declare that the research was conducted in the absence of any commercial or financial relationships that could be construed as a potential conflict of interest.
